# A Multiparametric Study of Internalization of Fullerenol C_60_(OH)_36_ Nanoparticles into Peripheral Blood Mononuclear Cells: Cytotoxicity in Oxidative Stress Induced by Ionizing Radiation

**DOI:** 10.3390/ijms21072281

**Published:** 2020-03-26

**Authors:** Anna Lichota, Ireneusz Piwoński, Sylwia Michlewska, Anita Krokosz

**Affiliations:** 1 Department of Molecular Biophysics, Faculty of Biology and Environmental Protection, University of Lodz, 90-236 Lodz, Poland; 2 Department of Materials Technology and Chemistry, Faculty of Chemistry, University of Lodz, 90-236 Lodz, Poland; 3 Laboratory of Electron Microscopy, Faculty of Biology and Environmental Protection, University of Lodz, 90-236 Lodz, Poland; 4 Department of General Biophysics, Faculty of Biology and Environmental Protection, University of Lodz, 90-236 Lodz, Poland; 5 Department of Biophysics of Environmental Pollution, Faculty of Biology and Environmental Protection, University of Lodz, 90-236 Lodz, Poland

**Keywords:** fullerenol uptake, accumulation, ionizing radiation, mitochondrial membrane potential

## Abstract

The aim of this study was to investigate the uptake and accumulation of fullerenol C_60_(OH)_36_ into peripheral blood mononuclear cells (PBMCs). Some additional studies were also performed: measurement of fullerenol nanoparticle size, zeta potential, and the influence of fullerenol on the ionizing radiation-induced damage to PMBCs. Fullerenol C_60_(OH)_36_ demonstrated an ability to accumulate in PBMCs. The accumulation of fullerenol in those cells did not have a significant effect on cell survival, nor on the distribution of phosphatidylserine in the plasma membrane. However, fullerenol-induced depolarization of the mitochondrial membrane proportional to the compound level in the medium was observed. Results also indicated that increased fullerenol level in the medium was associated with its enhanced transport into cells, corresponding to its influence on the mitochondrial membrane. The obtained results clearly showed the ability of C_60_(OH)_36_ to enter cells and its effect on PBMC mitochondrial membrane potential. However, we did not observe radioprotective properties of fullerenol under the conditions used in our study.

## 1. Introduction

Nanomaterials (NMs) are structure components with at least one dimension less than 100 nm. NMs should possess superior properties with enhanced performance that typical materials do not have [1]. Nanoparticles (NPs) are created due to van der Waals forces, electrostatic interactions, hydrogen bonds, as well as capillary bridges. The ability of NPs to assemble and interconnect strongly depends on their physical and chemical characteristics and their surface properties, including size, shape, ratio of electric charges and hydrophobicity, and last, but not least, on the concentration and the presence of functional groups [2,3]. These factors, as well as ability to interact with superficial cellular receptors also determine the rate and mechanism of entry of NMs into a cell [4].

Fullerene C_60_ is a nanomaterial consisting of 60 carbon atoms connected by sp^2.5^-bonds. Due to those bonds, it has a pseudo-aromatic structure resulting from delocalization of π-electrons over the carbon core. This structure allows easy reaction with oxygen free radicals [4,5,6]. It was shown that pristine C_60_ fullerene was non-toxic at low concentrations, and it was able to penetrate cellular plasma membrane. It has also been shown to exhibit antioxidant properties [7]. Fullerene increases the activity of antioxidant enzymes and prevents deleterious effects of oxidative stress by direct reactive oxygen species (ROS) scavenging [8]. The C_60_ fullerene nanoparticle does not exert any genotoxic effect on human lymphocytes. C_60_ reduces the genotoxic effect of doxorubicin (DOX) in normal human lymphocytes [9]. New fullerene derivatives can also be used for upconversion luminescence (UCL) and magnetic resonance imaging (MRI). That may be of great importance in “image-guided therapy” [10]. Fullerenes may be introduced anywhere in the body and they accumulate in specific organs, such as the liver, kidneys, and the spleen. Scientists suggested that fullerenes were able to pass into the blood from the gut and could be transported with blood [11,12]. Studies have shown that fullerenes affected the order of membrane lipids and induced membrane damage by lipid peroxidation. It was demonstrated that they accumulated in lysosomes of oyster hepatopancreas cells. The disposition of carbon NPs in biological systems is independent on the site of contact between NPs and an organism. It is related to the passage across cell membrane. Fluorescence quenching showed that C_60_(OH)_18–22_ accumulated in outer regions of the membrane bilayer, whereas C_60_ was found in deeper regions of the bilayer [13,14]. Other water-soluble fullerene derivatives, such as the dicarboxyfullerenes, concentrate in mitochondria or in endosome- or lysosome-like vesicles [15].

**Figure 1:**
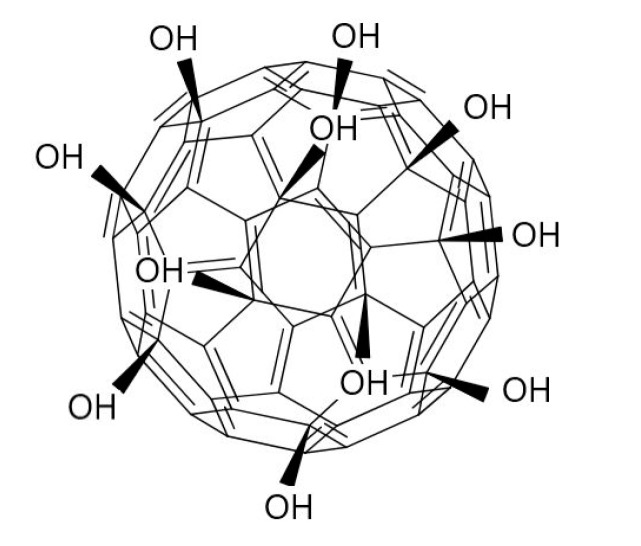
The structure of fullerenol C_60_(OH)_x_.

Fullerenols are bioactive compounds, polyhydroxylated water-soluble derivatives of fullerenes, a third natural allotropic variation of carbon [5,16,17]. The chemical structure of fullerenol C_60_(OH)_x_ is presented in Figure 1. The solubility of fullerenol in water increases with the increasing number of hydroxyl groups [18]. Fullerenols and malonic acid fullerene species are two major groups of functional fullerene derivatives possessing effective protective antioxidative properties both in vitro and in vivo, and having also antitumor potential and antimetastatic activity [19,20,21]. The results of the study carried out by Yin et al. (2009) [22] indicated that C_60_(C(COOH)_2_)_2_, C_60_(OH)_22_, and Gd@C_82_(OH)_22_ could protect cells against H_2_O_2_-induced oxidative damage, stabilized the mitochondrial membrane potential, and reduced intracellular ROS production. Due to their hydrophilic properties and the ability to scavenge free radicals, fullerenol could provide a valuable alternative to conventional pharmacological agents [17]. The effect of polyhydroxylated fullerenol NPs on adult male Wistar rats treated with DOX was investigated by Jacevic et al. (2017) [23]. The authors reported that fullerenol increased the survival rate, body and liver weight, as well as decreased the level of thiobarbituric acid reactive substances (TBARS), antioxidative enzyme activity, and hepatic damage score in DOX-treated rats. Fullerenol exhibits excellent biocompatibility and has other advantages, including high aqueous solubility and neutral pH. Its susceptibility to further modifications makes fullerenol a promising agent for medical applications [24,25]. Previous experimental results suggested that fullerenol C_60_(OH)_24_ induced ion permeability of a model membrane via the formation of ion pores or conductive defects. A preference for cations over anions was also observed [26].

The action of ionizing radiation on biological systems is mostly associated with the formation of ROS by the radiolysis of water. ROS action results in oxidative damage of proteins, lipids, deoxyribonucleic acid (DNA) and, as a consequence, leads to loss of cell function and cell death [20,27,28]. Cell death is related to damage to nuclear DNA and formation of DNA double-strand breaks (DSBs). Damage to the DNA can be caused directly by radiation or indirectly, through mediation of radicals, peroxides, and superoxides [29]. In order to reduce adverse radiation effects on biological systems, radioprotective compounds are researched [27]. Fullerens and their hydroxyl derivatives are a group of compounds studied for their possible application as antioxidants and radioprotective compounds [30,31].

The aim of the present study was to investigate pathways and processes involved in the uptake, translocation, and accumulation of fullerenol C_60_(OH)_36_ in peripheral blood mononuclear cells (PBMCs). We chose PBMCs as they are commonly used in toxicological studies. Moreover, we decided to perform some additional studies: measurement of fullerenol size and zeta potential, and the influence of fullerenol on the ionizing radiation-induced damage to PMBCs.

## 2. Results

### 2.1. Characterization of Fullerenol Nanoparticles

**Figure 2:**
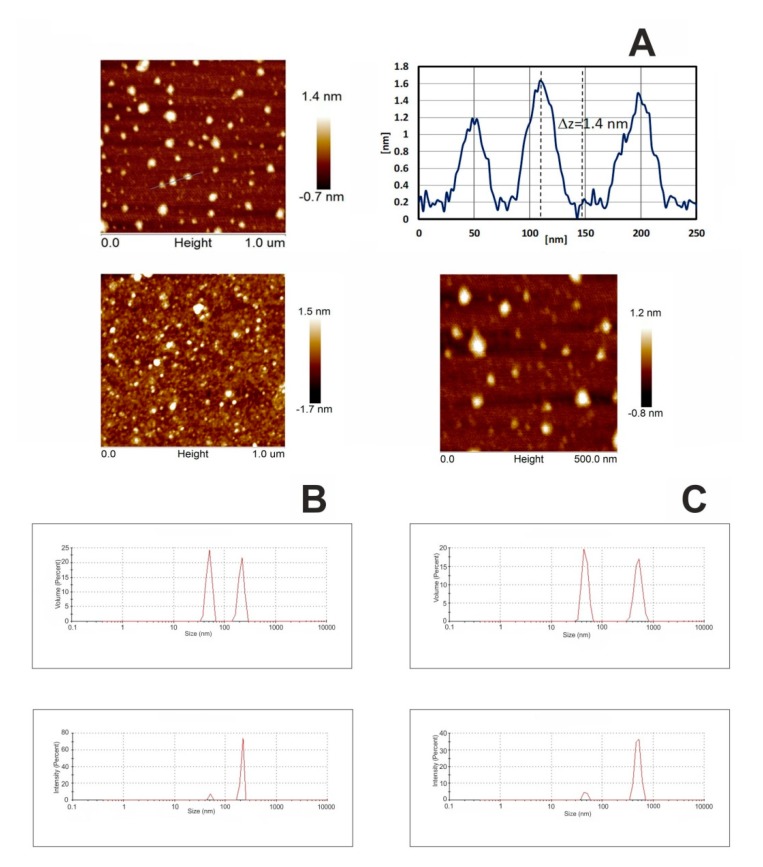
Atomic force microscopy (AFM) topography and surface profile of fullerenol. C_60_(OH)_36_ was dissolved in water and precipitated on mica substrate (**A**). Dynamic light scattering (DLS) size distribution of the C_60_(OH)_36_ in water (**B**) and in 0.02 M phosphate buffer pH 7.4 (**C**), showing the intensity and volume distributions of fullerenol-water and fullerenol-PBS preparations. Intensity-weighted average values were used to determine the hydrodynamic size, while volume distribution data was used to determine relative amounts of nanoparticles (NPs). Graphs in Figure 2B,C were produced by the Zetasizer Nano-ZS software.

Fullerenol NPs were characterized using atomic force microscopy (AFM) and dynamic light scattering (DLS) techniques. Figure 2A presents morphology images of fullerenol deposited by droplet precipitation on cleaved mica substrate, from aqueous solution, acquired using AFM. Measurements were performed after complete evaporation of the solvent. The AFM study revealed some randomly distributed agglomerates visible as white, round-shaped objects with the approximate local height of 1.4 nm. The size of analyzed fullerenol in solution was determined with the use of dynamic light scattering, while the particle surface potential (zeta potential) was determined by electrophoretic light scattering. Particle size distribution of fullerenol in aqueous solution and in 0.02 M phosphate buffer (pH 7.4) is presented in Figure 2B,C, respectively.

**Figure 3:**
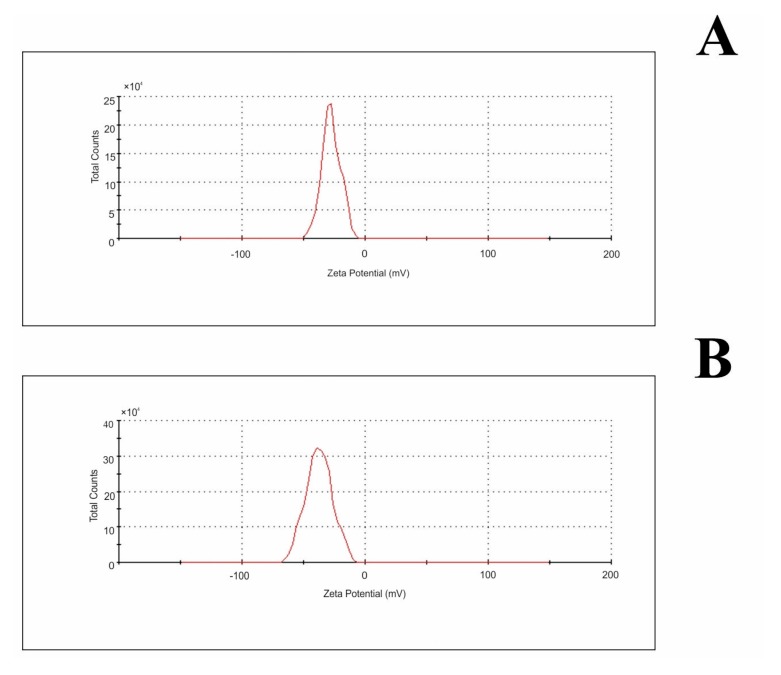
Zeta potential distribution of fullerenol NPs in water (**A**) and 0.02 M phosphate buffer pH 7.4 (**B**).

DLS data indicated that NPs with an average diameter of 40 nm and 110 nm predominated in C_60_OH_36_ aqueous suspensions, suggesting the presence of hydrated clusters formed by several fullerene molecules. Zeta potential measurements, evaluated by electrophoretic mobility of NPs (Table 1; Figure 3), indicated that hydrated NPs of fullerene acquired a negative surface charge (−27.5 mV for water and −37.4 mV for buffer). Negative values of zeta potential suggest that fullerene NPs were surrounded by a well-organized layer of hydrogen-bonded water molecules (a stable hydrophilic shell), which promoted a negative surface charge and prevented interaction with similar neighboring molecules.

### 2.2. Direct Evidence of Fullerenol Internalization by Confocal Microscopy and Flow Cytofluorometry

**Figure 4:**
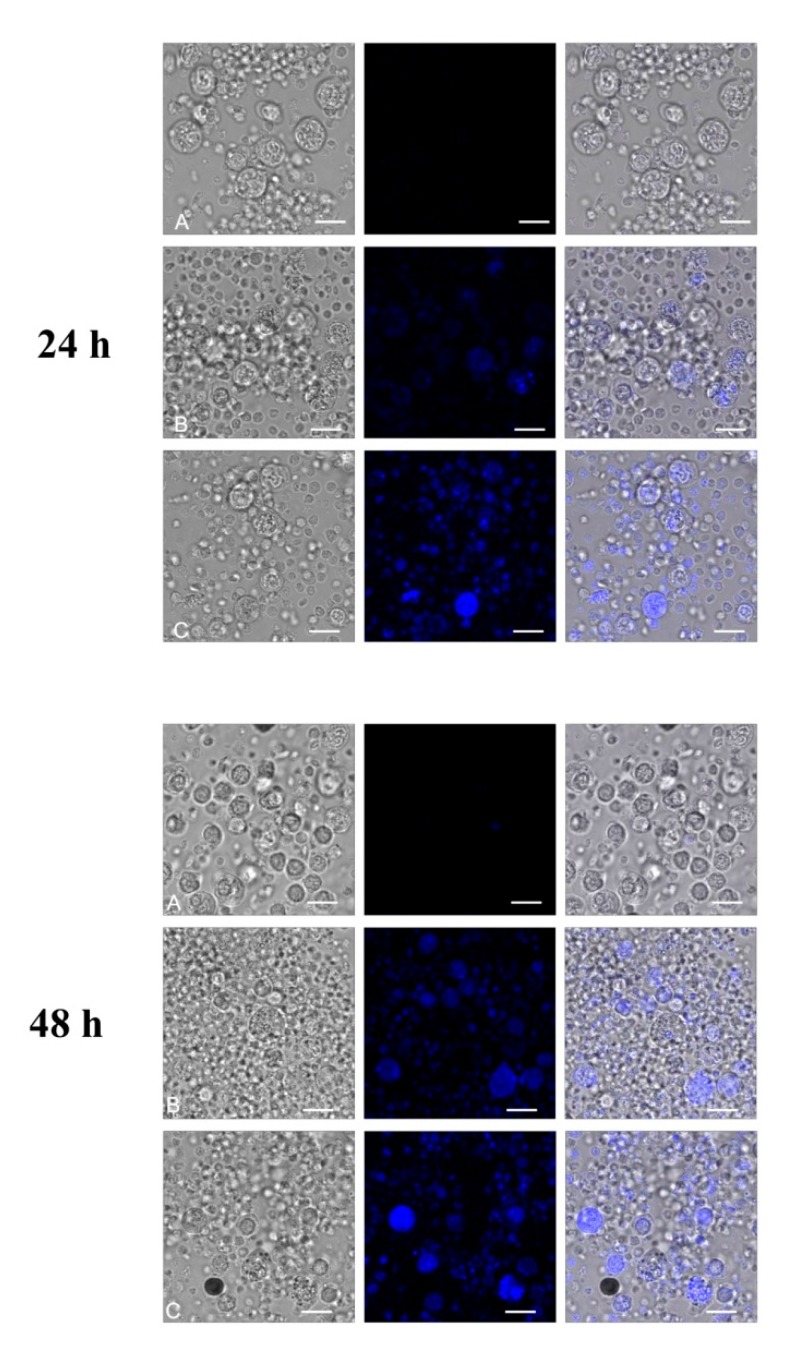
Confocal microscopy images of peripheral blood mononuclear cells (PBMCs) after 24 h and 48 h of incubation with fullerenol (37 °C, 5% CO_2_ atmosphere, 100% relative humidity). To analyze nanoparticle internalization, the following excitation and emission wavelengths were used: 405 nm for excitation and the range of 420–530 nm for emission. A, Control (without fullerenol); B, 75 mg/L; C, 150 mg/L. Bar = 10 μm.

**Figure 5:**
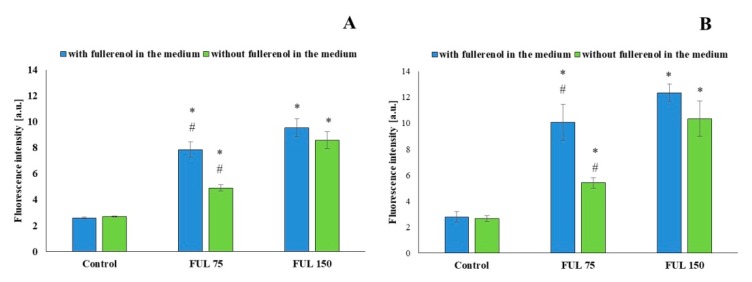
Fluorescence of fullerenol NPs inside PBMCs after 24 h (**A**) and 48 h (**B**) of incubation with fullerenol (37 °C, 5% CO_2_ atmosphere, 100% relative humidity). Blue bars—fullerenol was not washed out, green bars—fullerenol was washed out. Asterisks are used to mark values that are statistically different in comparison with control values (* *p* < 0.05), hashes—values that are statistically different in comparison with samples containing fullerenol at the concentration of 150 mg/L (# *p* < 0.05). All experiments were repeated 10–12 times.

Confocal microscopy techniques (Figure 4) were used to analyze the internalization of NPs into PBMCs after 24-h and 48-h incubation with fullerenol at the concentrations of 75 mg/L and 150 mg/L. We used the fullerenol property for autofluorescence at 345 nm excitation wavelength and at 470 nm emission wavelength in aqueous solution (own studies). Figure 5 shows that uptake of nanoparticles was concentration-dependent. More efficient internalization of nanoparticles was observed after incubation of PBMCs with NPs at the higher concentration. Internalization of fullerenol NPs was still observed when fullerenol was removed from the cell suspensions by centrifugation and fresh medium was added.

**Figure 6:**
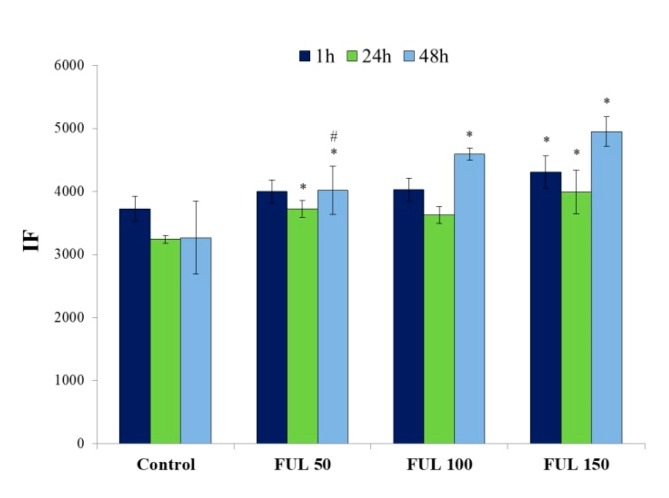
Quantitative measurements of fullerenol in PBMCs were carried out by flow cytometry in time-dependent mode (flow cytometer FACS, 355 nm laser); after 1 h, 24 h, and 48 h of incubation with NPs (37 °C, 5% CO_2_ atmosphere, 100% relative humidity). Bars represent mean values with standard deviations. Asterisks are used to mark values that are statistically different in comparison with control values (* *p* < 0.05), hashes—values that are statistically different in comparison with samples containing fullerenol at the concentration of 150 mg/L (^#^ *p* < 0.05). All experiments were repeated 3–6 times.

Flow cytofluorometry was used to determine the extent of adsorption of fullerenol (50–150 mg/L) on the cell surface and/or the compound internalization after 1 h, 24 h, and 48 h of incubation. Figure 6 shows that, as in the case of confocal microscopy, the uptake of nanoparticles by the cells depended on the concentration and incubation time. The highest cellular uptake of NPs was observed for their concentration of 150 mg/L.

### 2.3. Effects of Fullerenol on the Viability of PBMCs

**Figure 7:**
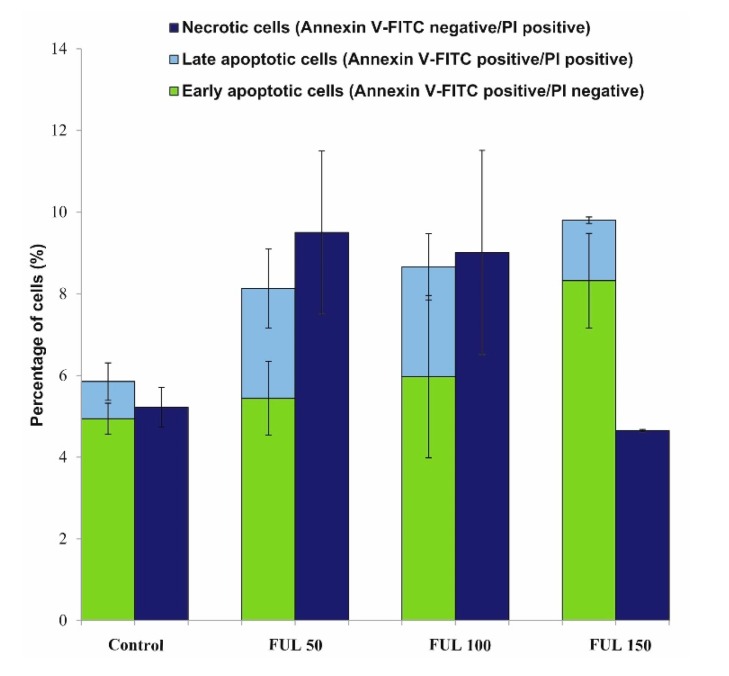
Cytometric analyses of cell viability for cells stained with PI and Annexin V-FITC. PBMCs were incubated in the absence or presence of fullerenol from 50 to 150 mg/L for 24 h (37 °C, 5 % CO_2_ atmosphere, 100% relative humidity). The differences between control and fullerenol-treated cells were not statistically significant.

Annexin V is a protein demonstrating a high affinity for phosphatydylserine (PS). Externalization of PS occurs at early stages of apoptosis, which can be detected by Annexin V staining. Annexin V-FITC/propidum iodide (PI) double staining is useful for distinguishing between viable cells (unstained) and apoptotic cells (stained with Annexin V-FITC), and necrotic cells (stained with PI). Figure 7 shows quantitative changes in PBMCs after 24 h incubation with fullerenol (50–150 mg/L). According to those results, C_60_(OH)_36_ does not affect cell viability compared to the control (viable cells ~85%).

### 2.4. The Impact of Fullerenol on Human Peripheral Blood Mononuclear Cells under Radiation-Generated Oxidative Stress

**Figure 8:**
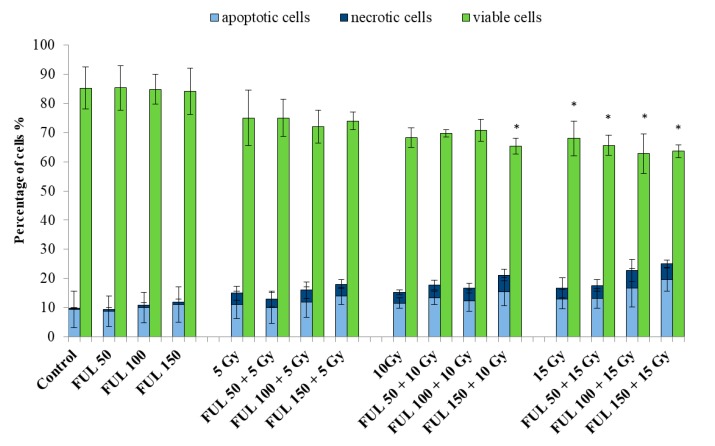
Viability of PBMCs irradiated (5, 10, and 15 Gy) in the absence or presence of fullerenol C_60_(OH)_36_ (50–150 mg/L) after 24-h-post-irradiation incubation (37 °C, 5% CO_2_ atmosphere, 100% relative humidity). Preincubation with fullerenol lasted 1 h. The cells were stained with PI and calcein-AM (c-AM). Bars represent mean values with standard deviations. Asterisks are used to mark values that are statistically different in comparison with control values (* *p* < 0.05). All experiments were repeated 3–6 times.

Radioprotective properties of fullerenol were also tested. PBMCs were irradiated (5, 10, and 15 Gy) in the absence and presence of fullerenol C_60_(OH)_36_ and labeled with PI/ calcein-AM (c-AM) after 24 h from the end of irradiation. No significant apoptotic changes were detected in the control, after incubation with fullerenol alone, and at 5-Gy and 10-Gy irradiation. In response to radiation, statistically significant changes were observed for the combination of 10 Gy radiation with the highest concentration of fullerenol. The results obtained by means of this method were similar to those for Annexin V-FITC/PI staining. The number of apoptotic cells increased with increasing fullerenol concentration and a dose of radiation (Figure 8).

It is possible that structural disorders of plasma membrane (demonstrated in previous studies) [32] caused by fullerenol C_60_(OH)_36_ were responsible for the effect of this substance observed during irradiation of PBMCs. Previous studies [33] proved that fullerenol lowered the post-radiation hemolysis, potassium efflux, and oxidation of thiol groups in human anucleate erythrocytes. However, fullerenol enhanced toxic effects of radiation toward membrane acetylcholinesterase.

**Figure 9:**
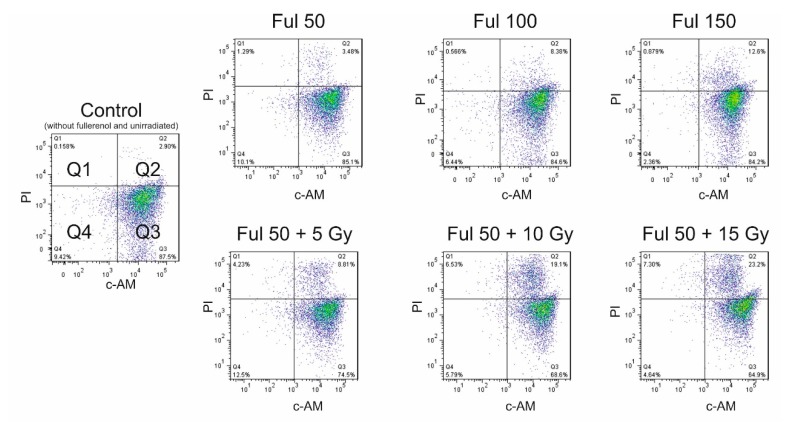
Representative cytograms of PBMCs after incubation with fullerenol C_60_(OH)_36_ (0–150 mg/L) for 24 h (37 °C, 5% CO_2_ atmosphere, 100% relative humidity), or preincubated with fullerenol (50 mg/L) for 1 h, and then irradiated with the doses of 5, 10, and 15 Gy in the presence of fullerenol. Viability was assessed 24 h after the end of irradiation. Q1—necrotic cells; Q2—apoptotic cells; Q3—viable cells; Q4—unstained cells. The cells were double stained with PI and calcein-AM (c-AM).

Figure 9 shows cytograms of PBMCs cultured for 24 h with fullerenol C_60_(OH)_36_ (50–150 mg/L). The cells were irradiated with 5-, 10-, and 15-Gy doses with/without fullerenol. The histogram shows the intensity of green fluorescence (c-AM) in the X-axis and of red fluorescence (PI) in the Y-axis. Cytograms obtained from the flow cytometry analysis showed that dose of 15 Gy caused a decrease in the count of viable cells.

### 2.5. Assessment of Mitochondrial Membrane Potential after Incubation with Fullerenol

**Figure 10:**
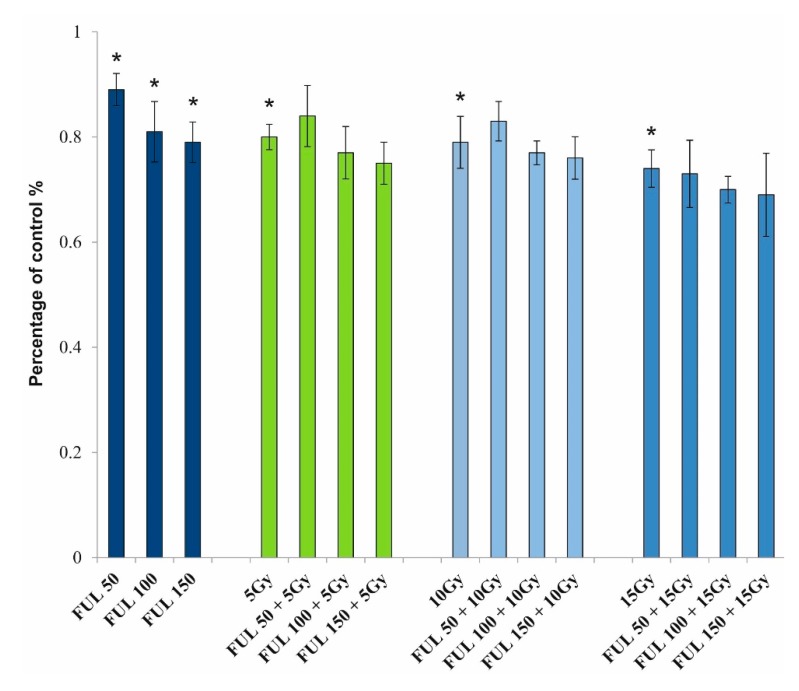
Mitochondrial membrane potential of PBMCs. PBMCs were incubated with fullerenol C_60_(OH)_36_ (50, 100, and 150 mg/L) for 24 h (37 °C, 5% CO_2_ atmosphere, 100% relative humidity) and irradiated with the doses of 5, 10, and 15 Gy. The presented values are the fraction of untreated cells (control) for which the value was set at 1.0. Bars represent mean values with standard deviations. Asterisks are used to mark values that are statistically different in comparison with control values (* *p* < 0.05). All experiments were repeated 4 times.

The fluorescent probe JC-1 was used to determine the effect of fullerenol on PBMCs with or without radiation-generated oxidative stress. Figure 10 shows changes in mitochondrial membrane potential of PBMCs depending on the concentration of fullerenol (50–150 mg/L) and irradiation (5, 10, and 15 Gy) after 24-h incubation (37 °C, 5% CO_2_ atmosphere, 100% relative humidity). Fullerenol reduced mitochondrial membrane potential in PBMCs in a concentration-dependent manner. At its highest concentration (150 mg/L) fullerenol showed a similar effect to the 10-Gy ionizing radiation, reducing mitochondrial potential down to approximately 80% of the control value (measured in cells that were not treated with fullerenol nor irradiated). Fullerenol at none of the applied concentrations (50–150 mg/L) showed protective activity against ionizing radiation, nor intensified the effect of irradiation.

## 3. Discussion

Numerous hydroxyl groups present on the surface of fullerenol enable the formation of multiple hydrogen bonds to various biomolecules, such as protein domains of the cell membrane and hydrophilic lipid heads. Fullerenol can be adsorbed onto cytoskeletal proteins and this interaction may be used as a drug transport mechanism [32,33,34,35]. Furthermore, fullerenol exhibits antioxidative properties [36,37].

More and more carbon NPs enter the environment and living organisms [38,39,40]. Therefore, it is important to identify the mechanisms involved in NPs’ transport, and assess their impact on cells, including blood cells. Blood cells are a valuable research model, as they are commonly exposed to xenobiotics and are easy to separate.

This paper is the first to present results of the study clearly showing penetration of fullerenol C_60_(OH)_36_ NPs into cells and their adsorption on plasmatic membrane. Earlier reports [41,42] indirectly indicated adsorption and penetration of those polar NPs into cells. Molecular dynamics studies [43] also demonstrated a possible transport of fullerenol into cells.

Methods of confocal microscopy and flow cytometry were applied. Fluorescent properties of fullerenol were also used to directly demonstrate the penetration of highly hydroxylated fullerenol into cells. The obtained results indicated that C_60_(OH)_36_ entered PBMCs in the amount proportional to its concentration used and cell incubation time. This study also showed that at longer incubation time, intracellular accumulation of this NP prevailed over its adsorption on the external, hydrophilic surface of the cellular membrane (Figure 5). 

Our previous study demonstrated that fullerenol was able to adsorb on membrane surface, particularly in the vicinity of integral membrane proteins, such as transmembrane ionic pumps: ATPases K/Na, Mg, and Ca, irreversibly inhibiting the activity of those enzymes [32].

NPs can easily enter living cells by phagocytosis, macropinocytosis, caveolae-mediated endocytosis, or clathrin-mediated endocytosis [44,45]. Physicochemical properties of NPs (size, shape, surface charge, and surface chemistry) influence their uptake efficiency by cells [46]. Aggregation of NPs may be changed depending on the composition of media and functional coatings [47].

In this study, zeta potential measurements evaluated by electrophoretic mobility of NPs (Table 1; Figure 3) indicated that hydrated NPs of fullerene acquired a negative surface charge (−27.5 mV for water and −37.4 mV for phosphate buffer, pH 7.4). Negative values of zeta potential suggest that fullerenol NPs were surrounded by a well-organized layer of hydrogen-bonded water molecules (a stable hydrophilic shell), which promoted a negative surface charge and prevented interaction with similar neighboring molecules.

Existence of a layer of water molecules was confirmed by the results of DLS and AFM measurements. The mean diameter of fullerenol NPs in water solution was 40–110 nm. However, AFM results revealed that fullerenol particles, which stripped off the aqueous shell, were approximately 2 nm in diameter.

Electrostatic interactions between fullerenol molecules and between those molecules and the cell membrane may be largely responsible for the biological effect of C_60_(OH)_36_ in cells. That was confirmed by observations made by other researchers, who reported opposed effects of fullerenol, even with the same number of OH groups bound to the C_60_ fullerene core [48,49]. Similarly, this study indicated the absence of a direct linear correlation between the cellular fullerene content and its concentration in the medium (Figure 5).

Fullerenols C_60_(OH)_x_—water-soluble derivatives of fullerenes—are currently being intensively studied in the context of their possible application in biomedicine [50]. Due to its hydrophilic properties and the ability to eliminate free radicals, fullerenol may in the future provide a solid alternative to currently used pharmacological methods in chemotherapy, treatment of neurodegenerative diseases, and radiobiology [51,52].

However, depending on the research protocol applied, fullerenol may also act as prooxidant [53]. This dualistic nature of fullerenols may contribute to the future development of some new biomedical and environmental applications of these agents [54,55,56].

This study demonstrated that fullerenol C_60_(OH)_36_ at the concentrations up to 150 mg/L showed no toxic effect on PBMCs. No statistically significant differences in the induction of apoptosis or necrosis were observed between fullerene untreated and treated PBMCs (Figure 7, Figure 8, Figure 9). It is worth noting that the toxic effect of fullerenol was assessed using two methods for apoptosis detection. The first method monitored externalization of phosphatidylserine on the outer leaflet of cell membrane (Annexin V-FITC), and the second determined changes in the intracellular calcium level (calcein-AM staining). Consistent results were obtained from both methods, indicating an absence of toxic effect of fullerenol C_60_(OH)_36_ over the analyzed concentration range on PBMCs.

Bacchetta et al. [57] showed that shape and size were important factors influencing carbon NPs uptake by cells and tissues. Investigations imply that cytotoxicity is strongly correlated with the size and shape of NPs, physical parameters of cell culture media, and biochemical interactions with the cell occurring following internalization [58]. The results of this study indicate that fullerenol particles of 40–110 nm in diameter possessing a negative surface charge are absorbed by PBMCs and do not initiate cell death, even at the concentration of 150 mg/L.

It was observed, however, that fullerenol caused depolarization of the mitochondrial membrane potential in PBMCs in a concentration-dependent manner (Figure 10). Yang et al. [59] studied isolated mitochondria and demonstrated the same effect of fullerenol C_60_(OH)_44_. Moreover, C_60_(OH)_44_ increased the permeability of the mitochondrial inner membrane to H^+^ and K^+^ and induced mitochondrial permeability. The effect was concentration-dependent and was inhibited by cyclosporin A and EDTA.

As ionizing radiation is known to cause depolarization of the mitochondrial membrane potential [60], and numerous reports indicated that fullerenols were effective scavengers of free radicals and exhibited antioxidative activity [33,61], this study analyzed the effect of fullerenol C_60_(OH)_36_ on the mitochondrial potential in irradiated PBMCs. Unfortunately, it was found that the presence of fullerenol failed to protect from depolarization of the mitochondrial membrane potential caused by ionizing radiation at doses of up to 15 Gy. Similarly, no protective effect of fullerenol on radiation-induced death of PBMCs was observed. PBMCs irradiated with 15 Gy demonstrated a reduced viability following a 24-h incubation started directly after irradiation. Simultaneous exposure of cells to fullerenol and irradiation showed that this NP failed to protect PBMCs from death.

Similar results were reported by Nowak et al. [62], who did not observe a protective action of fullerenol following a 24-h incubation, for the very high dose of 50 Gy. On the other hand, the electron spin resonance spectroscopy (ESR) studies confirmed that C_60_(OH)_24_ was a powerful radical scavenger for superoxide, hydroxyl, and lipid radicals. Those data suggested that C_60_(OH)_24_ was a mitochondrial protective antioxidant with direct radical scavenging activity and indirect antioxidant activity [61].

The free radical mechanism of ionizing radiation suggests that scavengers of free radicals should protect cellular structures against damage. It is possible, however, that depolarization of the mitochondrial membrane potential, as well as structural disorders of plasma membrane (demonstrated in previous studies) [32] caused by fullerenol C_60_(OH)_36_ were responsible for the effect of this substance observed during the irradiation of PBMCs. It is worth noting that the effects of fullerenol and ionizing radiation were neither synergistic, nor additive.

The mitochondrial membrane potential (ΔΨm) is a result of the difference in the concentration of ions, including H^+^, on both sides of the inner mitochondrial membrane. Therefore, it depends on the electrochemical potential gradient and the pH gradient. Calcium ions play an important role in the maintenance of homeostasis of ΔΨm because they control the opening of the mitochondrial permeability transition (MPT) and adenosine triphosphate (ATP) production. Depolarization of the inner ΔΨm enhances outflow of cations from and inflow of anions to mitochondria, wherein the outflow is not proportional, but exponentially dependent on ΔΨm [63]. Excessively high potential of the mitochondrial membrane favors the production of ATP, but also of ROS. Over-production of ROS leads to damage to the mitochondrial membrane, and further generation of ROS. A cell is able to tolerate some short-term changes of the mitochondrial membrane potential, but long-term ΔΨm disorders lead to cell death.

Ionizing radiation induces both cellular and mitochondrial oxidative stress, leading to the generation of ROS, as reviewed thoroughly in [64]. Despite the fullerenol-induced mitochondrial membrane depolarization observed in this study, this effect did not trigger apoptosis or necrosis of PBMCs (Figure 7, Figure 8). This finding may be explained by the ability of fullerenol to bind to membrane proteins, and by—at least partial—inhibition of outflow of cations from mitochondria, as a result of mitochondrial membrane depolarization. Irreversible binding of fullerenol to ion-dependent ATPases was confirmed in previous studies [32]. Fullerenol binding and inhibition of ionic transport through the outer mitochondrial membrane resulting from block of the voltage-dependent anion channel 1 (VDAC1) cannot be excluded either. It is known that reduced mitochondrial membrane potential predispose channels to transport some large ions, even up to 5 kDa [65].

Using density functional theory calculations, Wang et al. [66] investigated ROS (O_2_^•‒^, ^•^OH, and H_2_O_2_) scavenging mechanisms for C_60_ fullerenols and the relationship between fullerenol’s structure and radical-scavenging ability. The addition of ^•^OH to C_60_ yields ^•^C_60_OH with ΔG = –43.1 kcal × mol^−1^. Because the removal of one electron from C_60_ is endothermic, with ΔG = 36.6 kcal × mol^−1^ in water, direct addition should be an exclusively dominant pathway for C_60_ to scavenge ^•^OH. Despite the fact that ionizing radiation predominantly acts indirectly on cells, via ROS (including ^•^OH, O_2_^•‒^, and H_2_O_2_) production, no protective effect of fullerenol was observed in analyzed systems, which confirms the previous statement of direct interference between fullerenol and cellular structures.

Considering the size of fullerenol molecules determined in this study, and their tendency to form aggregates/conglomerates, the actual concentration of NPs may be insufficient to compete with ROS, despite a nominally high concentration.

## 4. Materials and Methods

The following components were used for the synthesis of fullerenol C_60_OH_36_: fullerene C_60_ 99.5% (SES Research, Houston, TX, USA), sodium hydroxide, methanol and hydrogen peroxide 30% (Avantor Performance Materials Poland SA, Gliwice, Poland) and Amberlit MB20 (Sigma-Aldrich, St. Louis, MO, USA). Peripheral blood mononuclear cells were obtained from leukocyte platelet buffy coat from healthy donors purchased from the Regional Centre of Blood Donation and Blood Treatment, Lodz, Poland. 

### 4.1. Chemicals and Reagents

Histopaque-1077, RPMI 1640 medium (pH 7.4), trypan blue (TB), (0.4% (*v*/*v*)), ethidium bromide (EB), acridine orange (AO), calcein-AM (c-AM), phosphate buffered saline (PBS) (0.02 M, pH 7.4), fetal bovine serum (FBS), streptomycin, and penicillin were obtained from Sigma-Aldrich (St. Louis, MO, USA). FITC-Annexin V Apoptosis Detection Kit II was obtained from BD Biosciences-Pharmingen. All other chemicals were of analytical grade and were purchased from POCh S.A. (Gliwice, Poland). Deionized water from the Milli-Q Plus system was used to prepare all solutions.

### 4.2. Methods

#### 4.2.1. Synthesis of Fullerenol Nanoparticles

Fullerenol C_60_(OH)_36_ was synthesized from fullerene C_60_ (99.5%). Sodium hydroxide, perhydrol (H_2_O_2_, 30%) and Amberlit MB20 were used for the synthesis of fullerene. Polyhydroxylated fullerene was obtained using the method described previously by Krokosz et al. [67]. First, fullerene C_60_ was reacted with solid NaOH and perhydrol (H_2_O_2_, 30%) at room temperature. Hydrolysis was carried out for 24 h. Fullerenol was precipitated with methanol. Subsequently, NaOH residues were removed by ion-exchange chromatography on AmberlitMB-20. The structure of the obtained C_60_OH_36_ was confirmed by IR spectrophotometry (NEXUS FT-IR spectrometer, Thermo Fisher Scientific, Waltham, MA, USA), ^1^H-NMR (Varian Gemini 200 MHz, Varian Inc., Palo Alto, CA, USA ), ^13^C-NMR (Bruker Avance III 600 MHz, Bruker Corp., Billerica, MA, USA), and mass spectroscopy MS-ESI (Varian 500 MS, Varian Inc., Palo Alto, CA, USA).

#### 4.2.2. AFM Characterization of Fullerenol

A drop of fullerenol C_60_(OH)_36_ water solution was deposited on freshly cleaved muscovite (mica) surface without any further pretreatment. Surface topography was analyzed with the use of atomic force microscopy (AFM)–Bruker Dimension Icon operating in ambient conditions in air. NSC35/Si_3_N_4_/AlBS (NT-MDT) cantilevers were used for the AFM tapping mode measurements. Typical scan sizes of the acquired images were: 0.5 µm × 0.5 µm, 1 µm × 1 µm, and 2 µm × 2 µm. The size of C_60_(OH)_36_ agglomerates deposited on mica was estimated on the basis of measurement of the height surface profile taken by the analytic tool in the Bruker NanoScope Analysis software (ver. 1.40, 2012, Bruker Corp., Billerica, MA, USA). 

#### 4.2.3. Measurement of Size 

The average diameters of fullerenol NPs in water and in buffer solutions were determined by dynamic light scattering (DLS) on Zetasizer Nano-ZS (Malvern Instruments, Malvern, UK). Samples were measured at 25 °C in water and 0.02 M phosphate buffer pH 7.4 in plastic cells DTS0012 (Malvern Instruments, Malvern, UK). The analysis was made using the Malvern Instruments software. The refraction factor was assumed to be 1.33, while the detection angle was 90°, and the wavelength was 633 nm.

#### 4.2.4. Measurement of Zeta Potential

Measurement of electrophoretic mobility of samples by dynamic light scattering was conducted based on the phase analysis of light scattering (Zetasizer Nano-ZS). Samples in electric field were prepared in capillary cells (DTS1061). Samples were measured at 25 °C in water and 0.02 M phosphate buffer pH 7.4. The zeta potential value was calculated directly from the Helmholtz–Smoluchowski equation using the Malvern software.

#### 4.2.5. Preparation of Peripheral Blood Mononuclear Cell Suspensions

Peripheral blood mononuclear cells (PBMCs) were separated from erythrocytes and granulocytes by density gradient centrifugation with Histopaque 1077 (at 800 RCF for 25 min). After rinsing with a separation medium, PBMCs were cultured in RPMI 1640 medium with 10% FBS, 2 mM L-glutamine, 10 mg/mL streptomycin and 1000 U/mL penicillin. For the experiments, PBMCs demonstrating viability above 95% were only used (the analysis with Trypan Blue 0.4%). The cells were incubated in sterile conditions in a Smart Cell incubator (37 °C, 5% CO_2_ atmosphere, 100% relative humidity).

#### 4.2.6. Fullerenol Treatment Conditions

Fullerenol C_60_(OH)_36_ from the stock solution (3 mg/mL) was added to cell suspensions to final concentrations of 50, 75, 100, or 150 mg/L. The solution of fullerenol was sonicated for 1 h before being added to the cell suspension.

#### 4.2.7. Measurement of Fullerenol Internalization by Confocal Microscopy

After the isolation and above-mentioned treatment of PBMCs with NPs, drops of samples were placed on a slide and covered with a cover glass. The Leica TCS SP8 confocal microscope (Leica-Microsystems, Wetzlar, Germany) featuring 20× and 63×/1.20 lenses (HC PL APO CS2 63×/1.40 OIL) was used for microscopic imaging. To analyze the uptake of NPs, the 405-nm LED was used, with excitation and emission parameters of 420–530 nm. The LAS X Software (Leica-Microsystems, Wetzlar, Germany) was used to examine the fluorescence intensity of NPs in 100 cells.

#### 4.2.8. Measurement of Fullerenol Internalization by Flow Cytofluorometry

Fullerenol exhibits fluorescence at 345-nm excitation wavelength and at 470-nm emission wavelength. In order to determine the adsorption of fullerenol on cell surface and/or internalization, flow cytofluorometry was used (flow cytometer FACS, LSR® II from Becton-Dickinson, San Jose, CA, USA; 355 nm laser). In each experiment, 10,000 events were counted after gating of viable cells. Median of fluorescence intensity was calculated for each cell population. Untreated cells were used as a control.

#### 4.2.9. Conditions of Irradiation

PBMCs were irradiated using the Stabilipan X-ray machine (Siemens, Germany) equipped with a 2-mm aluminum filter. Dosimetry was performed before irradiation of PBMCs on a 6-well plate. The radiation dose delivered to the plate was 1.75 Gy/min, 185 kV, and 10 mA. Cells were irradiated with doses of 5, 10, and 15 Gy (irradiation on a 6-well plate) with or without fullerenol. The final concentration of fullerenol C_60_(OH)_36_ in tested samples was 50–150 mg/L. Before irradiation, the cells were incubated with or without the analyzed compound for 1 h at 37 °C, in 5% CO_2_ atmosphere with 100% relative humidity. After irradiation, the samples were incubated for 24 h and 48 h at 37 °C in 5% CO_2_ atmosphere and 100% relative humidity.

#### 4.2.10. Cell Viability

Cell viability was determined using calcein-AM (green, viable cells) and propidium iodide (PI) (red, non-viable cells). Calcein-AM, an acetoxymethyl ester of calcein, is highly lipophilic and easily penetrates cell membrane. In viable cells, calcein, formed from calcein-AM by esterase, emits green fluorescence (excitation: 490 nm, emission: 515 nm). PI cannot penetrate a viable cell membrane. It is able to penetrate a damaged membrane of a dead (necrotic) cell. Following entering a non-viable cell, the compound interacts with DNA and emits red fluorescence (excitation: 535 nm, emission: 617 nm).

#### 4.2.11. Detection of Apoptosis

During apoptosis, the cell is characterized by loss of structural asymmetry of cell membrane, and unrestricted permeability. Phospholipid phosphatidylserine (PS) is translocated from the inner to the outer surface of the membrane. In order to assess the type of cell death induced by fullerenol C_60_(OH)_36_ (50–150 mg/L for 24 h), double staining with Annexin V-FITC and PI was used. The double staining was carried out using the FITC-Annexin V Apoptosis Detection Kit II (BD Biosciences-Pharmingen, San Jose, CA, USA). PBMCs were suspended in binding buffer and stained with Annexin V-FITC and PI, respectively, and incubated for 15 min at room temperature. The study was carried out using the FACS flow cytometer (LSR® II from Becton-Dickinson, San Jose, CA, USA).

#### 4.2.12. Detection of Mitochondrial Membrane Potential

Mitochondrial membrane potential (ΔΨm) is an important parameter of mitochondrial function. To determine the effect of fullerenol on PBMCs, the lipophilic cationic dye 5,5′,6,6′-tetrachloro-1,1′,3,3′-tetraethylbenzimi-dazolylcarbocyanine iodide (JC-1) was used. JC-1 can selectively change color from green fluorescence in the monomeric form to red fluorescence in the aggregated form. JC-1 is excited by 488-nm laser light wavelength and exhibits a fluorescence emission shift from green (529 nm) to red (590 nm). Cells stained with JC-1 were analyzed spectrofluorimetrically (Cary Eclipse Fluorescence Spectrophotometer–Varian).

#### 4.2.13. Statistical Analysis

All data are presented as mean ± standard deviation and asterisks are used to mark statistical significance of differences. Statistical analysis was performed using one-way and two-way ANOVA followed by the Tukey post hoc test for multiple comparisons. All statistics were calculated using the Statistica software (StatSoft, Tulsa, OK, USA). Values of *p* < 0.05 were considered statistically significant.

## 5. Conclusions

We have demonstrated that fullerenol C_60_(OH)_36_ could accumulate in peripheral blood mononuclear cells. The cellular accumulation of fullerenol had no significant effect on the survival of the cells, nor on the distribution of phosphatidylserine in the plasma membrane. However, fullerenol-induced depolarization of the mitochondrial membrane potential proportional to the concentration of NPs studied in the medium was observed. The obtained results have indicated that increased fullerenol concentration in the medium resulted in increased transport of this substance into the cells, reflected by its effect on the mitochondrial membrane. The obtained results have clearly shown the ability of C_60_(OH)_36_ to enter cells and the influence the mitochondrial membrane potential. However, we did not observe radioprotective properties of fullerenol under the conditions used in this study. These results may have important implications for the use of highly hydroxylated fullerenols in biomedical applications.

## Figures and Tables

**Table 1:** Characterization of C_60_(OH)_36_ hydrodynamic size as measured by zeta potential in water and 0.02 M phosphate buffer pH 7.4.

	Water	0.02 M Phosphate Buffer pH 7.4
zeta potential (mV) ± SD	−27.5 ± 1.0	−37.4 ± 3.0

## Abbreviations

AFM	Atomic force microscopy
AO	Acridine orange
ATP	Adenosine triphosphate
c-AM	Calcein acetoxymethyl ester
DLS	Dynamic light scattering
DNA	Deoxyribonucleic acid
DOX	Doxorubicin
DSB	Double-strand breaks
EB	Ethidium bromide
ESR	Electron spin resonance spectroscopy
FBS	Fetal bovine serum
FITC	Fluorescein isothiocyanate
JC-1	5,5′,6,6′-tetrachloro-1,1′,3,3′-tetraethylbenzimi-dazolylcarbocyanine iodide
MPT	Mitochondrial permeability transition
MRI	Magnetic resonance imaging
NMs	Nanomaterials
NPs	Nanoparticles
PBMCs	Peripheral blood mononuclear cells
PBS	Phosphate buffered saline
PI	Propidium iodide
PS	Phosphatydyl serine
ROS	Reactive oxygen species
TBARS	Thiobarbituric acid reactive substances
VDAC1	Voltage-dependent anion channel 1
